# Optionen und Herausforderungen für ein neues Strommarktdesign in der Krise

**DOI:** 10.1007/s10273-022-3288-z

**Published:** 2022-10-20

**Authors:** Axel Ockenfels

**Affiliations:** Wirtschafts- und Sozialwissenschaftliche Fakultät, Universität zu Köln, Universitätsstraße 22a, 50923 Köln, Deutschland

**Keywords:** D47, H12, H23

## Abstract

Die Politik scheint entschlossen, in die Gestaltung des Stromgroßhandelsmarktes einzugreifen, um Krisengewinne und -belastungen umzuverteilen. Einige Vorschläge in diese Richtung würden die Probleme jedoch verschärfen. Es werden Handlungsoptionen für ein neues Strommarktdesign aufgezeigt und die damit verbundenen Herausforderungen bei der Implementierung diskutiert.

## Abstract (en)

Policymakers seem determined to intervene in the design of the wholesale electricity market to redistribute crisis gains and burdens. However, some of the proposed interventions would exacerbate the problems. Options for a new electricity market design are outlined and the associated implementation challenges are discussed. The most promising market designs use tax gains from inframarginal power generation. However, these designs also face significant challenges due to widespread forward hedging in the market.

Durch die hohen Gaspreise können Kohlekraftwerke, Kernkraftwerke und erneuerbare Energien, die kein Erdgas verwenden, sehr hohe Gewinne machen. Der Grund ist, dass die Gaskraftwerke den Preis für den gesamten Markt bestimmen, wenn sie für die Versorgung benötigt werden. Dieser Mechanismus ist nicht das Ergebnis eines fehlerhaften europäischen Strommarktdesigns, sondern Folge von transparentem Handel in wettbewerblichen Märkten. Dies folgt aus zwei fundamentalen Prinzipien des Wettbewerbs: *Erstens* produziert kein Kraftwerk Strom zu einem Preis, der unter den variablen Kosten liegt, weil es sonst Verluste machen würde. Wettbewerb führt dazu, dass zu jedem Zeitpunkt nur die günstigsten Kraftwerke produzieren, und unter diesen günstigsten Kraftwerken muss der Preis folglich auch die variablen Kosten des teuersten Kraftwerks decken. *Zweitens* gilt, dass es in einem transparenten, wettbewerblichen Markt für ein homogenes Produkt (wie Strom) nur einen einzigen Marktpreis geben kann. Bei zwei oder mehr Preisen gäbe es Arbitragemöglichkeiten: Ein Händler könnte so lange billig kaufen und teuer weiterverkaufen, bis die Preise angeglichen sind (law of one price). Aus diesen beiden Prinzipien folgt, dass in einem Markt die stromproduzierenden Kraftwerke mit den höchsten variablen Kosten den Marktpreis bestimmen (Ockenfels, 2008 und 2009).

Manche Vorschläge zur Änderung des Strommarktdesigns laufen daher ins Leere oder sind gar kontraproduktiv. So könnte eine staatlich verordnete Preisobergrenze an den Strombörsen dazu führen, dass die Nachfrage steigt, das Stromangebot weiter sinkt und Anbieter zu weniger effizienten Handelsplattformen flüchten. Dies würde das zugrundeliegende Knappheitsproblem weiter verschärfen.

Dennoch gibt es Gründe darüber nachzudenken, wie „Zufallsgewinne“ oder „Windfall-Profits“ infolge des massiven Gaspreisanstiegs ausnahmsweise und zeitlich begrenzt umverteilt werden können. Unter normalen Umständen sind Windfall-Profits zwar dringend notwendig, um Investitionskosten zu decken und um Anreize für neue Investitionen zu geben (Cramton et al., 2013; Grimm et al., 2008; Ockenfels, 2008; Claeys et al., 2018). Allerdings spielt im europäischen Kontext nicht der Markt, sondern die Politik die zentrale Rolle für Markteintritt und -austritt bei Kohle, Kernkraft und teils auch bei erneuerbaren Energien. Vor allem erreichen aber die Strompreise in dieser Krise Größenordnungen, die sie zur unerträglichen Belastung für viele Stromverbraucher werden lassen. Welche Maßnahmen versprechen Entlastung? Kann die Neugestaltung der Regeln im Stromgroßhandel dabei helfen?

## Vorschlag für neue Regeln für den Stromgroßhandel

Ein Vorschlag Griechenlands ist, den Markt in zwei Segmente aufzuteilen, eines für die Erzeugung mit geringen variablen Kosten (etwa Wind) und eines für die teure Er-

* Diese Arbeit basiert auf Ockenfels (2022) und profitiert von einem engen Austausch mit vielen Experten, besonders Peter Cramton, Sebastian Kranz, Achim Wambach und Georg Zachmann, sowie mit Experten und Entscheidern in Ministerien, Regierung und der EU-Kommission. Ich bedanke mich für den wertvollen Input. Die Einschätzungen und Vorschläge spiegeln meine Ansicht wider. Ich bedanke mich auch beim European Research Council (GA No 741409 – EEC) und der Deutschen Forschungsgemeinschaft (EXC 2126/1– 390838866) für die Unterstützung meiner Forschung.

**Prof. Dr. Axel Ockenfels** lehrt an der Fakultät für Wirtschafts- und Sozialwissenschaften der Universität zu Köln und ist Experte für Marktdesign und Verhaltensforschung.

zeugung (etwa Gas). Dies würde für sich genommen jedoch nicht viel ändern, da die kostengünstigen Produzenten in ihrem Segment denselben Marktpreis wie in einem einzigen Markt durchsetzen könnten. Der Grund ist, dass alle verfügbaren Kraftwerke im günstigen Segment laufen, wann immer das teure Segment benötigt wird. Das günstige Segment produziert also an seiner Kapazitätsgrenze, sodass der Marktpreis in diesem Segment bei Wettbewerb nicht durch die variablen Kosten, sondern durch die Nachfrage determiniert wird – und im Ergebnis dem Marktpreis im teuren Segment entspricht. Daher wird in dem griechischen Modell zusätzlich vorgeschlagen, dass die kostengünstigen Erzeuger kostenbasiert und nicht marktgerecht entlohnt werden – was letztlich auf die Auflösung großer Teile des Strommarktes hinauslaufen würde. Die damit teils gravierenden Probleme werden von Maurer et al. (2022) beschrieben.

Um das zu vermeiden, hat z. B. Sebastian Kranz von der Universität Ulm vorgeschlagen, den für die kostengünstige Stromerzeugung gezahlten Preis zu begrenzen, sodass die Vorteile des Marktes zumindest teilweise greifen können (Kranz, 2022). Doch auch dieser Vorschlag verzerrt den Preismechanismus. Beispielsweise würde die Nachfrageseite durch die Preiskappung ineffizient viel Strom nachfragen, und Stromanbieter wären versucht, andere Verkaufskanäle zu finden, was dann weitere Interventionen nach sich ziehen müsste.

Eine alternative Handlungsoption ist, alle Einnahmen aus der Nicht-Gas-Stromerzeugung zu besteuern, und zwar nur in den Zeiten, in denen Gaskraftwerke benötigt werden und Strom liefern (Ockenfels, 2022). Die Steuer darf dabei den Nettopreis nicht unter die variablen Kosten der Produktion drücken, damit die Produktion der Nicht-Gas-Kraftwerke nicht gestoppt wird. Da die Steuer nur dann erhoben wird, wenn Gaskraftwerke Strom liefern, kann die Steuer die durch den hohen Gaspreis induzierten hohen Erlöse – die „inframarginalen Renten“ – aller anderen stromproduzierenden Kraftwerke effektiv abschöpfen. Dies kann unabhängig davon geschehen, welche Verträge der jeweiligen Stromproduktion zugrunde liegen und wo der Handel stattfindet. Gleichzeitig bleiben der Marktmechanismus und alle Anreize, Strom anzubieten und den Gasverbrauch zu senken, voll erhalten: Weil die preisbestimmenden Gaskraftwerke nicht besteuert werden, wird der Marktpreis nicht beeinträchtigt. Dennoch kommt es zu Steuereinnahmen, die in einer Weise zur Entlastung der Stromverbraucher verwendet werden sollten, die den Marktmechanismus nicht verzerren.

Die Europäische Kommission scheint derzeit einen ähnlichen Ansatz zu präferieren: In dem am 1. September 2022 publik gemachten „Non-paper on Emergency Electricity Market Interventions“ wird ein Preiscap auf inframarginal produzierten Strom im Spotmarkt vorgeschlagen. Der Vorschlag entspricht konzeptionell und vom Ergebnis her weitgehend dem oben beschriebenen Mechanismus.[1] Tatsächlich handelt es sich nicht um einen Preiscap im Sinne einer Begrenzung des von der Nachfrage bezahlten Strompreises. Vielmehr sollen die Erlöse, die bei einem Preis über dem Cap entstehen, im Nachgang zum Spotmarkt „wegbesteuert“ werden.

Die Bundesregierung hat sich am 3. September 2022 in einem Maßnahmenpaket ebenfalls für eine Begrenzung von „Zufallsgewinnen“ ausgesprochen. Dabei soll analog zu den genannten Vorschlägen eine „Erlösobergrenze für Anlagen der Stromerzeugung mit geringer Kostenbasis“ festgelegt werden und der „Differenzbetrag zwischen Großhandelspreis und Erlösobergrenze“ abgeführt werden.

## Ungelöste Herausforderungen

Falls Gewinne und Belastungen mit Hilfe des Stromgroßhandels umverteilt werden sollen, spricht viel dafür, eine Variante des vorgestellten Vorschlags in den Blick zu nehmen. Ob aber das Strommarktdesign überhaupt die richtige Stellschraube ist, hängt davon ab, ob die damit verbundenen Herausforderungen zufriedenstellend gelöst werden können, die sich bei der Implementierung ergeben. Das ist noch unklar.

Das größte Problem der Besteuerung oder der Kappung inframarginaler Erlöse besteht darin, dass Spotmarktpreise nicht notwendigerweise die Gewinne der Produzenten determinieren. Stromerzeuger verkaufen den Strom oft auf Termin, d. h. auf Märkten, die dem Spotmarkt vorgelagert sind, um Preisrisiken zu mindern. Die dort erzielten Preise können deutlich unter den Spotmarktpreisen liegen – besonders, wenn diese massiv unterschätzt wurden, weil die Verträge und die Preise schon vor der Krise besiegelt wurden. Dann aber machen die Stromproduzenten deutlich geringere Gewinne als die Preise im Spotmarkt nahelegen würden und die Abschöpfung mutmaßlicher Gewinne kann Produzenten in die Verlustzone bringen.[2]

Terminmarktgeschäfte sind eine große Herausforderung für eine Politik der Umverteilung via Strommarktdesign, zumal in der Regel nicht nachvollzogen werden kann,

1 Allerdings entscheidet beim Kommissionsvorschlag der Regulierer oder die Politik, welches Kraftwerk als inframarginal klassifiziert wird, während im zuvor beschriebenen Mechanismus der Markt für jede Stunde endogen ermittelt, welches Kraftwerk besteuert wird.

2 Analoge Herausforderungen gibt es für erneuerbare Energien, deren Erlöse durch Einspeisevergütungen, Marktprämien, PPAs, CfDs und andere Instrumente abgesichert wurden.

welcher Marktteilnehmer welche Termingeschäfte getätigt hat. Eine theoretisch denkbare Lösung wäre, dass Produzenten eine „ungerechtfertigte“ Abschöpfung mutmaßlicher Gewinne auf die relevanten nachgelagerten Stufen weiterwälzen können, auf denen die Windfall-Profits tatsächlich anfallen. Dies wäre nicht ganz anders als die Idee, es Gasversorgern zu erlauben, die erhöhten Gaseinkaufspreise auf die Nachfrage weiterzuwälzen, die noch von niedrigen Preisen aus früheren Verträgen profitieren. Praktisch dürfte das jedoch schwierig umzusetzen sein. Zum Beispiel wird der auf Terminmärkten verkaufte Strom in der Regel nicht auf einzelne Kraftwerke heruntergebrochen: Stromanbieter poolen bei ihren Angeboten den Strom typischerweise aus unterschiedlichen Kraftwerken und vertraglich zugesicherten Lieferungen. Die für die Steuer nötige Differenzierung, welcher Strom mit welcher Kraftwerkstechnologie produziert wurde, ist dann nicht mehr möglich.

Da neu abgeschlossene Terminkontrakte bereits die hohen Preise reflektieren, könnte man alternativ über eine über die Zeit schrittweise ansteigende Abschöpfung der inframarginalen Renten im Stromgroßhandel nachdenken. Auch ist es denkbar, bei der Schätzung der Erlöse eines Kraftwerks die im Stromsektor „üblichen“ Zeitpunkte der Vermarktung mit den jeweiligen Preisen zu berücksichtigen, beziehungsweise die relevanten Informationen direkt bei den Produzenten zu erfragen. Diese und andere Ideen würden jedoch zu Verzerrungen führen, sodass Unternehmen im Vergleich zu den tatsächlich angefallenen Windfall-Profits zu wenig oder zu hohe Steuern zahlen müssen.

Ein weiteres potenzielles Problem ist die Verlagerung von Stromangeboten in andere Regionen oder auf andere Plattformen, die Windfall-Profits unangetastet lassen oder weniger stark belasten. Wenn beispielsweise der Preiscap speziell im Day-Ahead-Market umgesetzt werden soll, wie es der Kommissionsentwurf nahezulegen scheint, könnten Stromanbieter dem Cap leicht entgehen, indem sie auf andere Plattformen (OTC, Terminmärkte, Regelenergiemärkte) ausweichen. Auch die Verlagerung von Stromangeboten ins Ausland wäre möglich, wenn entsprechende Transportmöglichkeiten bereitstehen. Ausweichmanöver können eingedämmt werden, wenn (1) die Erlöse jeglicher Produktion gemäß Spotmarktpreis abgeschöpft werden, unabhängig davon, wo der Strom verkauft und hin geliefert wird, und wenn (2) die europäischen Mitgliedstaaten ihre Maßnahmen koordinieren.

Bei einer Abschöpfung inframarginaler Renten könnte es aus Produzentensicht auch vorteilhaft sein, Kraftwerke mit höheren variablen Kosten einzusetzen, wenn die Erlöse aus Kraftwerken mit geringeren variablen Kosten zu hoch besteuert oder zu stark limitiert werden. In diesem Fall würde die Steuer bzw. das Preislimit die Markteffizienz beeinträchtigen und den Strom sogar teurer machen, weil sie die Merit Order durcheinanderbringt. Durch geeignete Erlösgrenzen bzw. Steuersätze sollte sich das Problem aber vermindern lassen.

Alternativ lohnt es darüber nachzudenken, zur Verbesserung der Anreize der Anbieter und zur Vereinfachung der Intervention die gesamte Stromproduktion zu besteuern, wobei die Kraftwerke jedoch die Möglichkeit bekommen, ihre variablen Kosten von der Steuer abzuziehen. Im Ergebnis würden die marginalen Kraftwerke auch bei diesem Mechanismus (fast) keine Steuer zahlen, sodass der Marktmechanismus weitgehend funktionsfähig bleibt. Der Nachteil dieses Vorschlags wäre, dass dies eine Schätzung bzw. Überprüfung der abgesetzten Kosten durch Regulierungsbehörden erfordern würde.

## Diskussion

Es gibt unterschiedliche Möglichkeiten, die Krisengewinne und -belastungen umzuverteilen, auch ohne Interventionen in das Strommarktdesign. Die Politik scheint aber entschlossen, in die Gestaltung der Stromgroßhandelsmärkte einzugreifen. Einige Vorschläge in diese Richtung würden die Probleme jedoch verschärfen. Am vielversprechendsten sind Vorschläge, die es durch die Besteuerung inframarginaler Renten bzw. durch eine Erlösbegrenzung bei inframarginalen Kraftwerken im Prinzip ermöglichen, die Verteilungsziele ohne Beeinträchtigung der Marktkräfte zu erreichen. Allerdings müssen vor einer Implementierung noch gewichtige konzeptionelle Probleme gelöst werden.

Eine Übergewinnsteuer, bei der die hochaggregierten Gewinne auf einer dem Spotmarkt weit nachgelagerten Stufe ermittelt und besteuert werden, verfolgt ebenso das Ziel Windfall-Profits im Strommarkt abzuschöpfen. Dieser Ansatz besitzt Vor- und Nachteile. Der Vorteil der Übergewinnsteuer ist, dass direkt die Gewinne anvisiert werden, während im Stromgroßhandel zunächst nur inframarginale Erlöse anvisiert werden, die aufgrund von Termintransaktionen nicht direkt mit den Gewinnen korrespondieren. Ein Nachteil ist, dass die Übergewinnsteuer zu buchhalterischen Steuerausweichmanövern führen dürfte, die im Stromgroßhandel so nicht möglich sind (siehe Fuest (2022) für fundamentale Bedenken gegen eine Übergewinnsteuer).

Welcher Weg der bessere ist, hängt davon ab, ob und wie gut sich die ökonomischen, rechtlichen und politischen Herausforderungen bei einer Implementierung jeweils lösen lassen. Denkbar ist auch, dass ein Mittelweg zwischen der stundenscharfen Abschöpfung im kurzfristigen Spotmarkthandel und der hochaggregierten Abschöpfung der langfristig erzielten Gewinne gangbar und empfehlenswert ist. So ist es z.B. vorstellbar, dass die schrittweise Einführung einer Besteuerung der inframarginalen Renten gemeinsam mit einer nachgelagerten Gewinnbesteuerung die gewünschte Abschöpfung präziser erreichen kann als die jeweiligen Maßnahmen für sich allein.

Eingriffe in das Strommarktdesign sollten sorgfältig mit anderen Maßnahmen abgewogen werden.[3] Falls sich die Politik für eine Intervention in das Strommarktdesigns entscheidet, sollte sie Vorschlägen zur Umverteilung der Krisengewinne und -belastungen folgen, die die Marktmechanismen nicht verwässern. Auch sollte über die Umverteilungsdiskussion nicht die Frage aus dem Blick geraten, wie das Strommarktdesign für seine ureigenen Aufgaben bei der Bepreisung und Allokation von Strom krisenfest gemacht werden muss.[4] Zum Beispiel spricht einiges dafür, dass sich mit einem klug ausgestalteten, liquiden Terminmarkt sowie mit Preisen, die auf regionale Besonderheiten reagieren können, extreme Knappheiten besser bewältigen lassen. Schnelle Experimente am offenen Herzen des Strommarktdesigns sind jedoch riskant und Fehler im Marktdesign sehr kostspielig, besonders inmitten einer Knappheitskrise.

3 Neben der Übergewinnsteuer gehören zu den anderen Maßnahmen, die ergänzend oder ersatzweise erwogen werden können, Vorschläge, wie der Energieeinkauf gegenüber Russland (z.B. Hausmann et al., 2022 sowie das „Non-paper on emergency wholesale price cap instruments for natural gas" der Kommission) und dem Rest der Welt (z.B. Boltz et al., 2022) strategisch besser aufgestellt werden kann, wie die Energienachfrage durch harte und softe Anreize (z.B. Berger et al., 2022) reduziert werden kann und wie der Gasmarkt in der Krise umorganisiert werden kann (z.B. das „Non-paper on TTF and representative benchmarks for wholesale natural gas" der Kommission).

4 Acatech (2020), Cramton (2017, 2022), Cramton et al. (2020), Joskow (2001), Ockenfels (2021) und Zachmann (2013) bieten dazu Analysen und Hinweise.

## References

[CR1] Acatech — Deutsche Akademie der Technikwissenschaften (2020), ESYS stellt Impulse für ein neues Marktdesign vor, https://www.acat.ech.de/allgemein/impulse-fuer-ein-neues-marktdesign/ (5. September 2022).

[CR2] Berger, S., A. Ockenfels und G. Zachmann (2022), The Behavioral Approach to Encouraging Household Participation in Gas Savings, *Working Paper*.

[CR3] Boltz, W., K. D. Borchardt, T. Deschuyteneer, J. Pisani-Ferry, L. Hancher, F. Lévêque, B., McWilliams, A. Ockenfels, S. Tagliapietra und G. Zachmann (2022), How to make the EU Energy Platform into an Effective Emergency Tool, *Policy Contribution, 10, Bruegel*.

[CR4] Claeys, G., G. Fredriksson und G. Zachmann (2018), The distributional effects of climate policies, *Bruegel Blueprint Series 28*, https://www.bruegel.org/sites/default/files/wp-content/uploads/2018/11/Bruegel_Blueprint_28_final1.pdf (5. September 2022).

[CR5] Cramton P (2017). Electricity market design. Oxford Review of Economic Policy.

[CR6] Cramton P (2022). Fostering Resiliency with Good Market Design: Lessons from Texas. ECONtribute Disussion Paper.

[CR7] Cramton P, Ockenfels A, Stoft S (2013). Capacity Market Fundamentals. Economics of Energy and Environmental Policy.

[CR8] Cramton P, Ockenfels A, Roth A E, Wilson R B (2020). Borrow Crisis Tactics to get COVID-19 Supplies to Where they are Needed. Nature.

[CR9] Fuest, C. (2022), Acht Gründe, warum eine Übergewinnsteuer keine gute Idee ist, *ifo Standpunkt, 237*, https://www.ifo.de/publikationen/2022/ifo-standpunkt/acht-gruende-warum-eine-uebergewinnsteuer-keine-gute-idee-ist (5. September 2022).

[CR10] Grimm V, Ockenfels A, Zoettl G (2008). Strommarktdesign: Zur Ausgestaltung der Auktionsregeln an der EEX. Zeitschrift für Energiewirtschaft.

[CR11] Hausmann, R., A. Łoskot-Strachota, A. Ockenfels, U. Schetter, S. Tagliapietra, G. Wolff, G. Zachmann (2022), Cutting Putin’s Energy Rent: „Smart Sanctioning“ Russian Oil and Gas, *Working Paper 05/2022, Bruegel*, und *CID Faculty Working Paper, 412, Harvard University*.

[CR12] Joskow P L (2001). California’s Electricity Crisis. Oxford Review of Economic Policy.

[CR13] Kranz, S. (2022), A Proposal for Capping Exploding Electricity Spot Market prices without Subsidies or Supply Reduction, *Economics and R—R posts*, http://skranz.github.io/r/2022/08/29/ProposalElectricitySpotMarketPrices.html und http://skranz.github.io/r/2022/09/02/InfraMarginalProfitsAndHedging.html (5. September 2022).

[CR14] Maurer, C., I. Schlecht und L. Hirth (2022), The Greek market design proposal would be the end of electricity markets as we know them, *Euractiv Media Network*, https://www.euractiv.com/section/electricity/opinion/the-greek-market-design-proposal-would-be-the-end-of-electricity-markets-as-we-know-them/ (5. September 2022).

[CR15] Ockenfels, A. (2008), Neun Beobachtungen zur Preisbildung im liberalisierten Strommarkt. Darf man seiner Intuition vertrauen?, in W. Löwer (Hrsg.), *Bonner Gespräch zum Energierecht* (Band 3), 9–29, V&R Unipress.

[CR16] Ockenfels, A. (2009), Probleme von Strompreiskontrollen aus volkswirtschaftlicher Sicht, in W. Löwer (Hrsg.), *Bonner Gespräche zum Energierecht* (Band 4), 25–42, V&R Unipress.

[CR17] Ockenfels A (2021). Marktdesign für eine resiliente Impfstoffproduktion. Perspektiven der Wirtschaftspolitik.

[CR18] Ockenfels, A. (2022), A simple proposal to skim electricity firm’s windfall profits, Euractiv Media Network, https://www.euractiv.com/section/energy/opinion/a-simple-proposal-to-skim-electricity-flrms-windfall-profits/ (8. September 2022).

[CR19] Zachmann, G. (2013), Electricity without borders: a plan to make the internal market work, *Bruegel Blueprint Series 20*, https://www.bruegel.org/book/electricity-without-borders-plan-make-internal-market-work (5. September 2022).

